# Neuroprotection of Tropical Fruit Juice Mixture via the Reduction of iNOS Expression and CRH Level in *β*-Amyloid-Induced Rats Model of Alzheimer's Disease

**DOI:** 10.1155/2020/5126457

**Published:** 2020-04-15

**Authors:** Theng Choon Ooi, Munirah Ahmad Munawar, Nur Hasnieza Mohd Rosli, Siti Nur Aqilah Abdul Malek, Hanisah Rosli, Farah Wahida Ibrahim, Norazrina Azmi, Hasnah Haron, Razinah Sharif, Suzana Shahar, Nor Fadilah Rajab

**Affiliations:** ^1^Center for Healthy Ageing & Wellness, Faculty of Health Sciences, Universiti Kebangsaan Malaysia, Jalan Raja Muda Abdul Aziz, 50300 Kuala Lumpur, Malaysia; ^2^Biomedical Sciences Programme, Faculty of Health Sciences, Universiti Kebangsaan Malaysia, Jalan Raja Muda Abdul Aziz, 50300 Kuala Lumpur, Malaysia; ^3^Center for Toxicology and Health Risk Studies, Faculty of Health Sciences, Universiti Kebangsaan Malaysia, Jalan Raja Muda Abdul Aziz, 50300 Kuala Lumpur, Malaysia; ^4^Faculty of Pharmacy, Universiti Kebangsaan Malaysia, Jalan Raja Muda Abdul Aziz, 50300 Kuala Lumpur, Malaysia

## Abstract

This study aimed to determine the effects of tropical fruit juice mixture (pomegranate, white guava, and Roselle) on biochemical, behavioral, and histopathological changes of *β*-amyloid- (A*β*-) induced rats. Formulation 8 (F8) of tropical fruit juice mixture was chosen for this present study due to its high phenolic content and antioxidant capacity. Forty Wistar male rats were divided into five groups: dPBS (sham-operated control), dA*β* (A*β* control), JPBS (F8 and PBS), JA*β* (F8 and A*β*), and IBFA*β* (ibuprofen and A*β*). F8 (5 ml/kg BW), and ibuprofen (10 ml/kg BW) was given orally daily for four weeks before the intracerebroventricular infusion of A*β* for two weeks. Histological analysis and neuronal count of hippocampus tissue in the Cornu Ammonis (CA1) region showed that supplementation with F8 was able to prevent A*β*-induced tissue damage and neuronal shrinkage. However, no significant difference in locomotor activity and novel object recognition (NOR) percentage was detected among different groups at day 7 and day 14 following A*β* infusion. Only effect of time differences (main effect of day) was observed at day 7 as compared to day 14, where reduction in locomotor activity and NOR percentage was observed in all groups, with F (1, 7) = 6.940, *p* < 0.05 and F (1, 7) = 7.152, *p* < 0.05, respectively. Besides, the MDA level of the JA*β* group was significantly lower (*p* < 0.01) than that of the dPBS group. However, no significant changes in SOD activity were detected among different groups. Significant reduction in plasma CRH level (*p* < 0.05) and iNOS expression (*p* < 0.01) in the brain was detected in the JA*β* group as compared to the dA*β* group. Hence, our current findings suggest that the tropical fruit juice mixture (F8) has the potential to protect the rats from A*β*-induced neurotoxicity in brain hippocampus tissue possibly via its antioxidant properties and the suppression of iNOS expression and CRH production.

## 1. Introduction

Alzheimer's disease (AD) is one of the most common neurodegenerative diseases in the elderly [1]. The clinical manifestations of AD include progressive loss of memory (dementia) and cognitive function, which are always accompanied by behavioral disorders [2]. The neuropathological hallmarks of AD include “positive” lesions such as the presence of amyloid plaques and cerebral amyloid angiopathy, neurofibrillary tangles (NFT), and glial responses and “negative” lesions such as loss of neurons and synapses [3]. While the senile amyloid plaques and NFT are the classical neuropathological lesions of AD, they likely only represent the surface of the pathological alterations that cause the cognitive decline associated with AD. Despite their cross-sectional nature, postmortem studies have enabled the staging on the progression of both amyloid and tangles which consequently lead to the development of diagnostic criterias that are being used worldwide currently [3].

Natural polyphenols have been reported to exert beneficial effects in preventing cardiovascular-related conditions but studies on its neuroprotective mechanisms are still lacking. Many of the biological effects of polyphenols have been attributed to their antioxidant properties, either through their reducing capacities per second or through their possible influences on intracellular redox status [4]. As such, polyphenols may protect cell constituents against oxidative damage and have been reported to limit the risk of various oxidative related degenerative diseases. The brain is particularly vulnerable to oxidative damage because it requires considerable amounts of energy and consumes a lot of oxygen in normal physiological conditions. Besides, the brain is also more susceptible to oxidative stress as compared to other organs due to the lower antioxidant defense capacity in the brain. The occurrence of oxidative stress in the brain is a key feature of sporadic Alzheimer's disease (AD) and manifests predominantly as lipid peroxidation [5]. The neuroprotective effects of many polyphenols rely on its ability to permeate the blood-brain barrier (BBB) and directly scavenge pathological concentration of reactive oxygen and nitrogen species and chelate transition metal ions [6]. Distinctive polyphenols were shown to have different scavenging activities and abilities to activate key antioxidant enzymes in the brain, thus breaking the vicious cycle of oxidative stress and tissue damage [7, 8]. Red-wine polyphenols with distinct structures have been documented to increase endothelial nitric oxide synthase (eNOS) while downregulating inducible nitric oxide synthase (iNOS) activity [9, 10]. Besides, tea polyphenols have been shown to increase neurological scores and reduce brain injury in oxyhemoglobin-induced subarachnoid hemorrhage mice [11]. Furthermore, the neuroprotective effects of polyphenols were also achievable by reducing neurological injury, which subsequently leads to the improvement in behavioral outcomes [12, 13].

Recently, accumulating evidence suggested that the classical hydrogen-donating antioxidant activity of polyphenols is unlikely to be the sole explanation for its cellular effects in vivo [4]. Polyphenols have exhibited several additional properties in a complex biological system, yet the mechanisms remain poorly understood. It is evident that polyphenols are potent bioactive molecules; therefore, a clear understanding of their precise mechanisms of action as either antioxidants or modulators of cell signaling is crucial to evaluate their potential as inhibitors of neurodegenerative diseases [4]. Besides, there are still limited studies on the neuroprotective effects of polyphenol-rich tropical fruits. Hence, this study aimed to determine the effects of tropical fruit juice mixture (pomegranate, white guava, and Roselle) on biochemical, behavioral, and histopathological changes of the A*β*-induced rat model of Alzheimer's disease. This is to explore the potential of the tropical fruit juice mixture to be developed as part of the therapeutic intervention in the prevention of chronic neurodegenerative diseases.

## 2. Materials and Methods

### 2.1. Preparation of Tropical Fruit Juice Mixture

Pomegranate, white guava, and Roselle juice were used to produce the tropical fruit juice mixture in this study. The fruit juices were mixed according to the 10 points centroid-simplex design modified method [14] with different ratios for each formulation. In this study, 4 different formulations, namely, formulation 7 (F7), formulation 8 (F8), formulation 9 (F9), and formulation 10 (F10) were used. F7 consisted of 1/2 distilled water, 1/3 pomegranate, 1/3 white guava, and 1/3 Roselle, while F8 consisted of 1/2 distilled water, 2/3 pomegranate juice, 1/6 white guava juice, and 1/6 Roselle juice. As for F9, it consisted of 1/2 distilled water, 1/6 pomegranate, 2/3 white guava, and 1/6 Roselle; while F10 was a mixture of 1/2 distilled water, 1/6 pomegranate, 1/6 white guava, and 2/3 Roselle. The concentrated fruit juice mixture was stored at −40°C before the analysis of antioxidant capacity (ferric reducing antioxidant power (FRAP) assay and 2, 2-diphenyl-1-picrylhydrazyl (DPPH) assay) and total phenolic content (TPC).

### 2.2. Analysis of Tropical Fruit Juice Mixture

The antioxidant capacity of the tropical fruit juice mixture was analyzed using FRAP assay, DPPH assay, and TPC analysis, with slight modification of the method proposed by Aziz et al. [15]. These assays were used to determine the best formulation in terms of antioxidant capacity and TPC content in the tropical fruit juice mixture. The best formulation was selected for further testing in the in vivo experiment.

### 2.3. Animals and Grouping

Forty Wistar male rats weighing 200 to 250 g were obtained from the Animal Unit, Faculty of Medicine, Universiti Kebangsaan Malaysia (UKM), following ethical approval from UKM Animal Ethics Committee (FSK/BIOMED/2013/NOR/20-MARCH/50-MARCH-2013-MARCH-2015). Rats were kept at constant room temperature (24°C) under a 12-hour light/dark cycle (lights on from 7 : 00 AM to 7 : 00 PM) and were acclimatized at least seven days before the experiment and grouped into five groups with eight rats per group. Group one was the sham-operated control group (dPBS) supplemented with distilled water. Group two was the A*β* control group (dA*β*) supplemented with distilled water. Group three (JPBS) was the sham-operated control group supplemented with tropical fruit juice mixture. As for group four (JA*β*) and five (IBFA*β*), they were A*β*-induced groups supplemented with tropical fruit juice mixture and ibuprofen (IBF), respectively.

### 2.4. Administration of Tropical Fruit Juice Mixture and Ibuprofen

**Figure 1:**
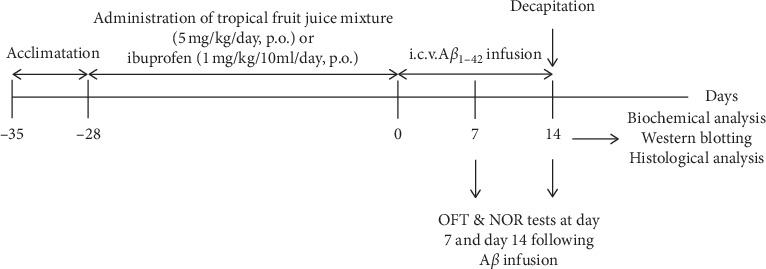
The experimental schedule of the study. i.c.v., intracerebroventricular; OFT, open field test; NOR, novel object recognition.

Tropical fruit juice mixture and IBF (Sigma-Aldrich Chemical, Saint Louis, USA) were given daily via oral gavage at a modified dosage of 5 ml/kg body weight [16] and 1 mg/kg/10 ml [17], respectively, for four weeks before A*β* infusion. For the dPBS group and dA*β* group, distilled water (5 ml/kg body weight) was given orally to the rats instead of tropical fruit juice mixture. The experimental schedule of the study is summarized in Figure 1.

### 2.5. Intracerebroventricular Surgery of Beta-Amyloid

Synthetic A*β*_1-42_ (0.1 mg/ml) was dissolved in phosphate-buffered saline (PBS) and incubated at 37°C for three days to allow the formation of A*β*_1-42_ aggregation. The rats were anesthetized using a mixture of ketamine, tiletamine, and xylazine (KTX) (0.1 ml/200 g) by intraperitoneal (i.p.) injection. A*β* was injected intracerebroventricularly (i.c.v.) using a bone microdrill, as described previously [18, 19]. A small incision was made on the head of the anesthetized rats to expose the skull. Then, one hole was drilled on the exposed skull (anteroposterior +1.2 mm from Bregma, mediolateral +2.0 mm, dorsoventral +4.0 mm) by using a stereotaxic apparatus. The cannula was affixed to the skull by using cyanoacrylate loctite glue (Loctite 454, USA). A subcutaneous pocket was prepared in the midscapular region of the back of the rats to receive the mini osmotic pump (ALZET, USA). The pump was then implanted in the subcutaneous pocket and was attached via polyvinylchloride tubing to the brain cannula. A*β*_1-42_ solution and vehicle (PBS) were spontaneously infused into the left lateral cerebral ventricle of the brain through the cannula by a mini osmotic pump for two weeks at a constant rate (0.5 *μ*l/hour), and the wound was stapled closed with wound clips (Reflex 7, USA). When the pump is properly placed, the tubing should have a generous amount of slack to permit free motion of the rats' head and neck. The rats require no restraint or handling during the delivery period.

### 2.6. Open Field Test

Rats were trained two days before drug and juice administration. The open field test (OFT) was performed before the novel object recognition (NOR) task. In this study, OFT was performed to measure locomotor activity in rats and exploration time in the central square of the arena [20]. The experiment was conducted in a soundproofed room illuminated with red light (20 watts) for easier recording of rats' activity. Each rat was placed in an open field arena (72 cm × 72 cm × 38 cm) and its exploration activity was recorded for five minutes. Recorded videos were analyzed, and the behaviors of rats were scored based on the frequency of line crossing, rearing, freezing, and time spent in the central square zone [21]. Locomotor activity was measured by the total frequency of line crossing and rearing.

### 2.7. Novel Object Recognition

The test was carried out with a slight modification of the method proposed by Broadbent et al. [22]. Each rat was acclimatized for 45 minutes in a room inside the cage and one minute in an empty arena before the experiment. NOR was divided into three phases: habituation, familiarization, and novel with a duration of seven days. The first phase was habituation for five minutes (day 1^st^ and 2^nd^), followed by the familiarization phase for 15 minutes from the 3^rd^ day until the 6^th^ day and lastly the novel phase for 10 minutes on day 7^th^. Rats were given two objects with similar shape and size during the familiarization phase but one of the familiar objects was replaced with a novel object during the novel phase. The object exploration was scored based on time spent to explore each object. Exploration was scored when the distance between the rat's noses was one cm from the placed object and there was movement of vibrissae [23]. Rats' preference for a novel object was expressed as the percentage of NOR exploration time compared to a familiar object.

### 2.8. Western Blot

Rats from each group were decapitated, and brains were rapidly removed using the aid of dried ice to maintain the necessary temperature. Following dissection, each hippocampus was weighed and homogenized with lysis buffer. Brain lysates containing protein were used for the preparation of loading samples. Western blot was performed with slight modification of the method by Mahmood and Yang [24]. Samples were separated on 10% sodium dodecyl sulfate-polyacrylamide gel electrophoresis (SDS-PAGE) and transferred to polyvinylidene difluoride (PVDF) membranes. By using a conventional method, the membrane was blocked with 4% bovine serum albumin (BSA) solution before washing with tris-buffered saline with 0.1% Tween 20 (TBST) solution 5 times (5 minutes for each wash). Then, the membrane was incubated with rabbit polyclonal anti-iNOS primary antibody (Abcam, USA; 1 : 1000 dilution) or rabbit monoclonal anti-*β* actin primary antibody (Abcam, USA; 1 : 1000 dilution) for 16 hours at 4°C and followed by 2 hours of incubation with HRP-conjugated anti-rabbit secondary antibody (Abcam, USA; 1 : 1000 dilution) at room temperature. The membrane was washed with TBST solution 5 times after every cycle of antibody incubation. Protein detection was conducted on the membrane by using Amersham enhanced chemiluminescence (GE Health Care, UK) and the Fusion FX7 documentation system (Vilber Lourmat, Germany).

### 2.9. MDA, SOD Activity, and Corticotropin-Releasing Hormone ELISA Assay Kit Determination

Brain MDA concentration and SOD activity, as well as plasma corticotropin-releasing hormone (CRH) level, were determined by using 96-well ELISA assay kits according to the manufacturer's instructions (Oxford Biomedical Research, USA; Cayman Chemical, USA; Cloud-Clone Corp, USA), respectively. Absorbance for each ELISA plate was measured at their respective wavelength by using a 96-well I-Mark™ microplate reader (Bio-Rad Laboratories, USA).

### 2.10. Histological Analysis of Hippocampus and Neuronal Count

The hippocampus of the brain was first sectioned and isolated by using brain matrices (Tedpella, USA). After fixing with 10% formaldehyde, the hippocampus tissue was dehydrated, embedded in paraffin, and sliced into 5 *μ*m-thick section before staining with Nissl stain [25]. Prepared slides were examined under a light microscope, and the neuronal count was performed within the selected area (100 *μ*m^2^) of each hippocampus tissue.

### 2.11. Statistical Analysis

Statistical analysis was performed using SPSS version 22 (IBM, USA). The data were expressed as mean ± standard error of the mean (S.E.M) while *p* value less than 0.05 was considered as statistically significant. For the behavioral test, repeated-measures ANOVA was carried out to determine the significant differences between different groups and days of *β*-amyloid infusion. On the other hand, the biochemical tests and the neuronal count data were analyzed by using one way ANOVA and followed by Tukey HSD post hoc test to determine the significant differences between different groups. For nonparametric data, the Kruskal–Wallis analysis was performed and followed by the Mann–Whitney *U* test.

## 3. Results and Discussion

### 3.1. Analysis of Tropical Fruit Juice Mixture

**Figure 2:**
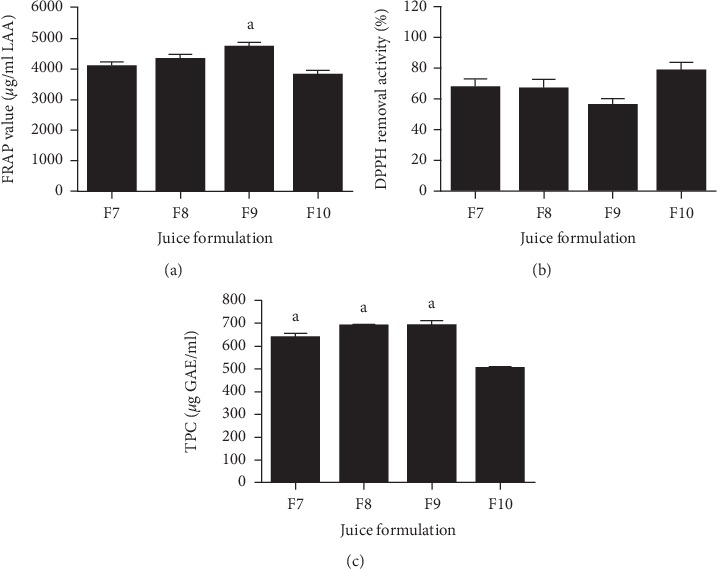
(a) FRAP value among different mixture juices formulation of three fruits. (b) DPPH radical removal activity among different mixture juices formulation of three fruits. (c) TPC among different mixture juices formulation of three fruits. Mixture juice 1/3 pomegranate + 1/3 white guava + 1/3 Roselle (F7), mixture juice 2/3 pomegranate + 1/6 white guava + 1/6 Roselle (F8), mixture juice 1/6 pomegranate + 2/3 white guava + 1/6 Roselle (F9), and mixture juice 1/6 pomegranate + 1/6 white guava + 2/3 Roselle (F10). ^a^Significant difference (*p* < 0.05) as compared to F10. All data are shown as mean ± standard error (*n *=* *4).

As shown in Figure 2(a), F9 (4725.25 ± 158.70 *μ*g/ml LAA) has the highest FRAP value and followed by F8 (4310.30 ± 185.40 *μ*g/ml LAA). On the other hand, F10 has the highest (78.51 ± 5.67%) DPPH radical removal activity among all the fruit juice formulations, as shown in Figure 2(b). For TPC (Figure 2(c)), the highest phenolic content was reported in F9 (690.27 ± 21.18 *μ*g GAE/ml). The TPC value of F8 (686.32 ± 7.32 *μ*g GAE/ml) was slightly lower than F9. However, no significant difference in the TPC value can be detected between these two formulations. Both F8 and F9 were reported to have higher TPC and FRAP values as compared to the other formulations. However, the DPPH radical removal activity of F8 was higher than F9. Hence, the best formulation chosen for this study was F8 due to its high phenolic content, FRAP value, and DPPH radical removal activity in overall.

### 3.2. Effects of Tropical Fruit Juice Mixture on A*β*-Induced Behavioral Changes

Tables 1 and 2 showed the locomotor activity in OFT and NOR percentage, respectively, between groups at day 7 and day 14 following A*β* infusion. Only effect of time differences (main effect of day) was observed at day 7 as compared to day 14, where reduction in locomotor activity and NOR percentage was observed in all groups (dPBS, dA*β*, JPBS, JA*β*, IBFA*β*), with F (1, 7) = 6.940, *p* < 0.05 and F (1, 7) = 7.152, *p* < 0.05, respectively. No significant difference in locomotor activity and NOR percentage was detected among different rat groups at these two time points.

### 3.3. Effects of Tropical Fruit Juice Mixture on A*β*-Induced Histological Changes

#### 3.3.1. Histological Analysis of the Cornu Ammonis Region in Hippocampus

**Figure 3:**
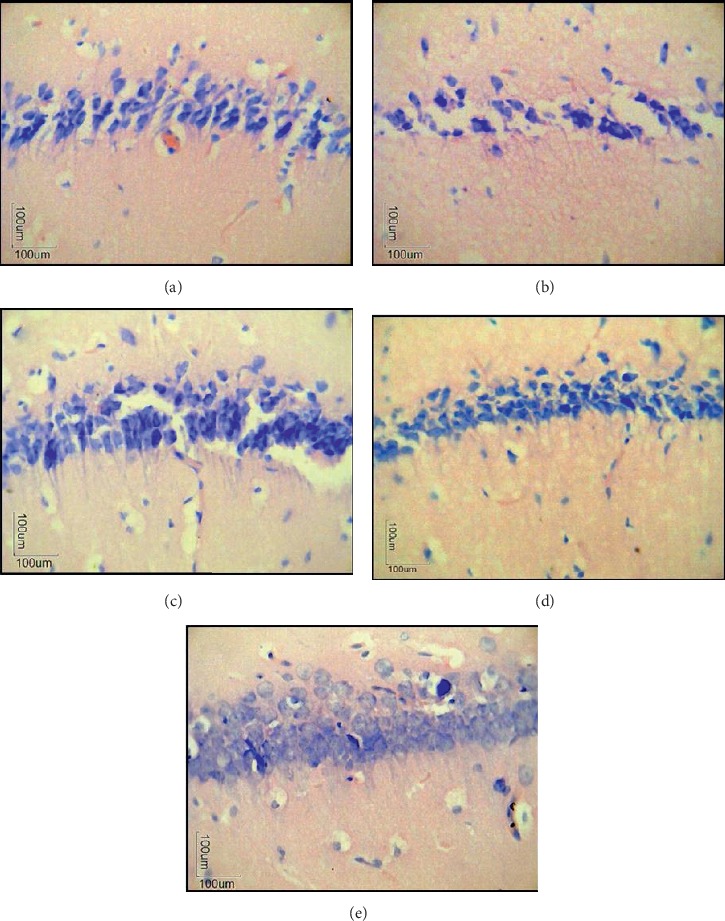
Histological analysis of the CA1 region in hippocampus brain tissue under Nissl staining (Cresyl violet). Slides were observed under ×400 magnification using a light microscope. (a) Sham-operated control (dPBS). (b) *β*-Amyloid control (dA*β*). (c) Mixture juice + PBS (JPBS). (d) Mixture juice + *β*-amyloid (JA*β*). (e) Ibuprofen + *β*-amyloid (IBFA*β*).

Figure 3 shows the cross-sectional histology of hippocampus tissue in the Cornu Ammonis (CA1) region under 400x magnification. In the sham-operated control group (dPBS), neuron cells were normal in shape and arranged in an orderly and compact manner (Figure 3(a)). Similar results were also seen in the JPBS group (Figure 3(c)). For the dA*β* group (Figure 3(b)), prominent tissue damage and shrinkage of neuronal cells were observed following the infusion of A*β* via i.c.v. Besides, neuron cells were not orderly arranged with the presence of gaps or spaces in between the cells. However, normal-shaped neuron cells were observed in the CA1 region of the hippocampus (Figure 3(d)) of the JA*β* group after A*β* infusion, indicating that supplementation with tropical fruit juice mixture was able to prevent A*β*-induced neuronal shrinkage and damage. On the other hand, ibuprofen-treated groups (IBFA*β* group) showed an order and compact arrangement of neuron cells (Figure 3(e)) following A*β* infusion.

#### 3.3.2. Neuronal Count in the Cornus Ammonis Region of Hippocampus

**Figure 4:**
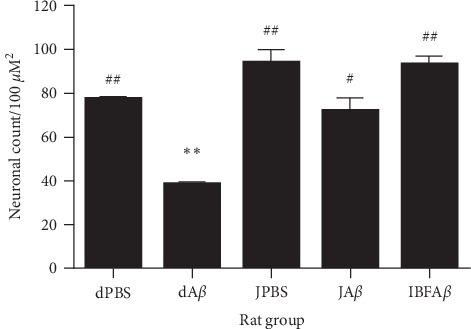
Neuronal count among different rat groups. Sham-operated control (dPBS), *β*-amyloid control (dA*β*), juice mixture + PBS (JPBS), juice mixture + *β*-amyloid (JA*β*), and ibuprofen + *β*-amyloid (IBFA*β*). ^*∗∗*^Significant differences (*p* < 0.01) as compared to the dPBS group. ^#^Significant differences (*p* < 0.05) as compared to the dA*β* group. ^##^Significant differences (*p* < 0.01) as compared to the dA*β* group. Data are presented as mean ± standard error with *n* = 8 per treatment group.

Figure 4 shows the results of the neuronal count of the CA1 region of the hippocampus. In normal conditions, supplementation with F8 did not cause any significant increment in neuronal count as compared to the dPBS control group. Infusion of A*β* by i.c.v. into the brain hippocampus of the dA*β* group (38.00 ± 2.00) caused a significant reduction (*p* < 0.05) in neuronal count as compared to the sham-operated control group (dPBS; 77.00 ± 1.00). However, supplementation with tropical fruit juice mixture or treatment with ibuprofen was able to attenuate the neurotoxic effects of A*β* infusion. Significant increment in neuronal count was observed in the JA*β* group (*p* < 0.05; 71.50 ± 6.50) and IBFA*β* group (*p* < 0.01; 93.00 ± 4.00) as compared to the dA*β* group (38.00 ± 2.00).

### 3.4. Effects of Tropical Fruit Juice Mixture on A*β*-Induced Biochemical Changes

#### 3.4.1. MDA Concentration and SOD Activity in Brain Homogenate

**Figure 5:**
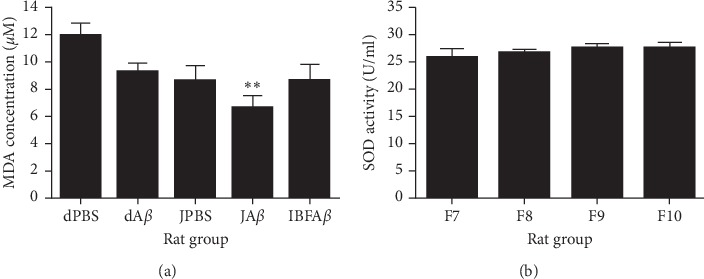
(a) MDA concentration in brain homogenates among different rat groups. (b) SOD activity in brain homogenates among different rat groups. Sham-operated control (dPBS), *β*-amyloid control (dA*β*), juice mixture + PBS (JPBS), juice mixture + *β*-amyloid (JA*β*), and ibuprofen + *β*-amyloid (IBFA*β*). ^*∗∗*^Significant difference (*p* < 0.01) as compared to the dPBS group. Data are presented as mean ± standard error with *n* = 8 per treatment group.

Infusion with A*β* did not cause any significant changes in MDA concentration in the brain homogenate of the dA*β* group as compared to the dPBS group (Figure 5(a)). Only rats supplemented with F8 (JA*β* group) showed a significant reduction (*p* < 0.01) in MDA concentration following A*β* infusion as compared to the dPBS group. On the other hand, no significant difference in SOD activity was detected in the brain homogenate of different rat groups, as shown in Figure 5(b).

### 3.5. iNOS Expression in Brain Homogenate

**Figure 6:**
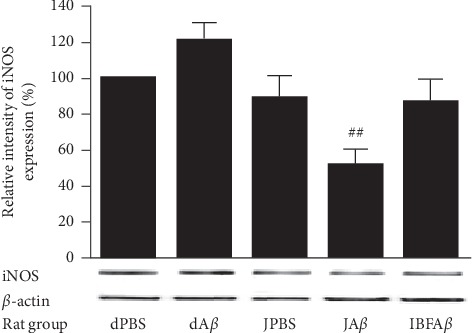
Intensity relative of iNOS expression in brain homogenates among different rat groups. Sham-operated control (dPBS), *β*-amyloid control (dA*β*), juice mixture + PBS (JPBS), juice mixture + *β*-amyloid (JA*β*), and ibuprofen + *β*-amyloid (IBFA*β*). ^##^Significant differences (*p* < 0.01) as compared to the dA*β* group. Data are presented as mean ± standard error with *n* = 3 per treatment group.

Figure 6 shows the expression level of iNOS in brain homogenates among different rat groups. Infusion of A*β* (dA*β* group) caused an increment in iNOS expression. Supplementation with F8 (JA*β* group) was able to suppress the induction of iNOS expression significantly (*p* < 0.01), as compared to the dA*β* group.

### 3.6. Concentration of CRH in Blood Plasma

**Figure 7:**
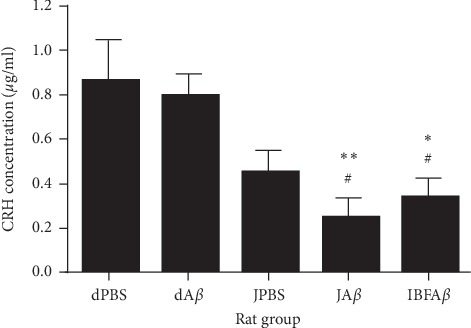
Plasma CRH concentration among different rat groups. Sham-operated control (dPBS), *β*-amyloid control (dA*β*), juice mixture + PBS (JPBS), juice mixture + *β*-amyloid (JA*β*), and ibuprofen + *β*-amyloid (IBFA*β*). ^*∗*^Significant differences (*p* < 0.05) as compared to the dPBS group. ^*∗∗*^Significant differences (*p* < 0.01) as compared to the dPBS group. ^#^Significant differences (*p* < 0.05) as compared to the dA*β* group. Data are presented as mean ± standard error with *n* = 8 per treatment group.

The plasma CRH concentration of different rat groups is shown in Figure 7. CRH concentration was significantly lower in the JA*β* group (*p* < 0.01; 0.244 ± 0.089 *μ*g/ml) and IBFA*β* group (*p* < 0.05; 0.336 ± 0.088 *μ*g/ml) as compared to the dPBS group (0.859 ± 0.189 *μ*g/ml). JA*β* and IBFA*β* group also demonstrated lower (*p* < 0.05) CRH concentration as compared to the dA*β* group (0.792 ± 0.097 *μ*g/ml) following A*β* infusion for 14 days.

## 4. Discussion

This study aimed to explore the neuroprotective effects of the tropical fruit juice mixture from three different polyphenol-rich fruits (pomegranate, white guava, and Roselle). The formulation F8 contains high phenolic content and antioxidant capacity as determined via FRAP, DPPH, and TPC analysis. These fruits have been documented to be rich in flavonoid, tannin, and phenolic acid [26–30] that help in increasing and sustaining antioxidant levels more effectively. This formulation was particularly rich in pomegranate which possesses high levels of antioxidant than other fruit juices and beverages [31, 32]. Some in vivo studies also suggested that pomegranate extract may be helpful in the prevention of AD [33]. On the other hand, ibuprofen, which is a nonsteroidal anti-inflammatory drug (NSAID), was used as a positive control for A*β*-induced AD-like conditions in this present study. Previously, ibuprofen has been shown to suppress senile/amyloid plaque pathology and inflammation in a transgenic AD mouse model [34].

Although the cause of AD remains unclear, A*β* accumulation in the brain was proposed to play a major role in the onset of this disease [35, 36]. Partial reproduction of AD neuropathological conditions and cognitive deficits can be achieved with pharmacological drug-induced approaches and A*β* overexpression [37]. In this study, A*β*-induced neurotoxicity (AD model) was chosen due to the ability of the A*β* protein-peptide to produce the symptoms and pathophysiology that mimics the slow evolution of AD in humans [19, 38]. The induction of neurotoxicity had successfully been achieved in this study since prominent tissue damage, shrinkage, and loss of neuronal cells were observed following the infusion of A*β* for 14 days consecutively. The previous study also demonstrated similar histological changes in the hippocampus region of rats following A*β* administration [26]. Histological analysis of the CA1 region in the hippocampus demonstrated that supplementation with the tropical fruit juice mixture can attenuate the neurotoxic effects of A*β*. Increase in the number of neuronal cells and the presence of neuronal cells with normal morphology following infusion of A*β* suggested that tropical fruit juice mixture possesses neuroprotective effects and has the potential to prevent the development and progression of neuronal diseases such as AD. Our current findings were further supported by the previous study where the A*β*-induced rat group supplemented with resveratrol was able to increase the neuronal count and reduced the neuronal damage caused by the neurotoxic effects of A*β* [26].

Central infusion of different types of A*β* such as A*β*_1-40_, A*β*_1-42_, or A*β*_25-35_ into the intracerebral ventricle or hippocampus produced memory impairment by forming oligomer assemblies in rodents, as confirmed by data from various behavioral tests [38–42]. However, our present study showed that A*β* alone did not cause any significant behavioral changes, as demonstrated via OFT and NOR tests after 7 days and 14 days of infusion. This could be due to the concentration of A*β* (0.1 mg/ml) being used in this current study to induce the neurotoxic conditions in rats was relatively lower as compared to others previous studies, which is possibly inadequate to cause any detectable changes in OFT and NOR tests within the study time frame [38, 42]. Hence, we were unable to investigate the protective effects of tropical fruit juice mixture against A*β*-induced behavioral changes in this present study under such circumstances.

In normal physiological conditions, redox status in cells is maintained by the antioxidant defense system [43]. However, excessive production of reactive oxygen species (ROS) may overwhelm the capacity of the antioxidant defense system, hence leading to the occurrence of oxidative stress. Oxidative stress is known to play an important role in the development of neurodegenerative diseases, such as Alzheimer's disease and Parkinson's disease [44]. It has been reported that the presence of A*β* can increase ROS production in neuronal cells, hence causing oxidative stress in the brain [45]. A previous study has demonstrated that high level of MDA was found in the brain tissue of AD [26]. On the other hand, lower SOD activity was observed in the A*β*-induced group as compared to the control group following A*β* infusion into hippocampal tissues [46, 47]. However, our current findings showed contrasting results where no significant changes in MDA concentration and SOD activity were detected between the dA*β* group and the dPBS group following the infusion of A*β*. It is possible that the rats might have developed compensatory mechanisms against A*β*-induced oxidative stress during the study period since we infused the rats with a relatively lower dose of A*β* for 14 days.

Our current findings showed that supplementation with tropical fruit juice mixture can reduce the MDA concentration in the brain of rats following A*β* infusion. However, it does not have any effects on SOD activity. Hence, we suggest that the neuroprotective effects of tropical fruit juice mixture against A*β*-induced neurotoxicity were solely due to its high phenolic content and antioxidant capacity present in the tropical fruit juice mixture. It has been suggested that antioxidant therapy may help in the prevention and treatment of neurodegenerative diseases [48, 49]. The presence of antioxidants derived from food sources has been suggested as a promoter for the endogenous antioxidant activity to reduce oxidative stress-aided process [50]. Food antioxidants derived from tropical fruit juice mixture of three different fruits (pomegranate, guava, and Roselle) contain different types of antioxidants that are beneficial to health, as proven previously [26–30]. Thus, we believed the composition of different fruits helps in increasing antioxidant levels more effectively.

Previous reports also demonstrated that A*β* protein is a potent neurotoxin that leads to the occurrence of neuroinflammation in the brain [51, 52]. The induction of neuroinflammation by A*β* infusion may induce the expression of iNOS in neuronal cells. iNOS, which is the inducible isoform of NOS catalyzes the conversion of L-arginine into one of the reactive nitrogen species (RNS) known as nitric oxide (NO) [53]. NO is a short-lived free radical that plays an essential role in various physiological functions, including inflammation. Overproduction of NO can be deleterious since it is cytotoxic, and it may cause tissue destruction [54]. Our current findings demonstrated that A*β* infussion can induce the expression of iNOS, and supplementation with the tropical fruit juice mixture can attenuate the induction of iNOS expression by A*β*. This observation was supported by previous findings where supplementation with polyphenols derived from lychee seed and resveratrol was able to reduce the iNOS expression in rats following A*β* induction [26, 55]. Thus, we suggest that the polyphenol-rich tropical fruit juice mixture may exert its neuroprotective effects by preventing the occurrence of neuroinflammation induced by A*β*.

Alterations in neuroendocrine function are also part of the clinical manifestations of AD. Previous studies have reported that A*β* deposition in AD transgenic mice and acute i.c.v. administration of A*β*_25-35_ peptides in rats were able to activate the hypothalamic-pituitary-adrenal (HPA) axis since soluble A*β* has been shown to activate the CRH neurons located in the paraventricular region of the hypothalamus [56–58]. However, our present study showed contrasting results where no significant changes in plasma CRH levels were detected in the dA*β* group following A*β* infusion as compared to the sham-operated control group. This is probably because the infusion of A*β* which is neurotoxic and can cause neuronal cell death, as demonstrated via histological analysis of the CA1 region of the hippocampus and neuronal count. The loss of CRH neurons may lead to the overall reduction in CRH production and secretion even in the presence of A*β* that has been shown to activate the HPA axis. Furthermore, inconsistent data with regards to the CRH level was reported previously, where the CRH level in the cerebrospinal fluid of AD subjects was found to increased, decreased, or remain unchanged in separate studies [59–61].

Disruption of the HPA axis has been consistently demonstrated in AD, usually preceding the cognitive decline [62]. It has been proved that hypothalamic dysfunction may drive the progression of AD. In a transgenic mouse model of AD, chronic stress can induce Tau pathology, neurodegeneration, and learning impairment, which can be blocked by CRH receptor 1 antagonists and enhanced by CRH overexpression [56]. Previous studies have demonstrated that *Crf* mutant animals showed a reduction in both A*β*- and Tau-related pathologies [63, 64]. Moreover, mutations in CRH receptor 1 have been proved to reduce Tau hyperphosphorylation and A*β* deposition in response to stress [65–67]. These data suggest that the activation of the HPA axis and subsequent increment in CRH concentration could contribute to brain dysfunction in AD. Results from this present study demonstrated that supplementation with the tropical fruit juice mixture was able to reduce the plasma CRH level in rats following A*β* infusion, suggesting that tropical fruit juice mixture has the potential to prevent the progression of AD by suppressing the activation of HPA axis. Besides that, the increase in the concentration of CRH was positively correlated to depression [68]. Thus, we suggest that tropical fruit juice may help in reducing depression and stress.

## 5. Conclusion

In conclusion, supplementation with formulation F8 of tropical fruit juice mixture (5 ml/kg body weight/day) can protect the CA1 region of the hippocampus of rats from prominent tissue damage, shrinkage, and neuronal loss following A*β* infusion for 14 days, possibly via its antioxidant properties and the suppression of iNOS expression and CRH production. Thus, the tropical fruit juice mixture (F8) has the potential to be developed as a neuroprotective agent in improving neuroinflammation and other neurodegenerative-related conditions.

## Figures and Tables

**Table 1:** Locomotor activity among different rat groups at day 7 and day 14.

Locomotor activity (ƒ)/time of A*β* infusion	dPBS group	dA*β* group	JPBS group	JA*β* group	IBFA*β* group
After 7 days	48.00 ± 7.62	57.33 ± 7.19	57.75 ± 10.41	63.22 ± 7.81	57.00 ± 6.17
After 14 days	30.50 ± 7.26^*∗*^	38.67 ± 3.92^*∗*^	38.38 ± 5.08^*∗*^	59.67 ± 6.47^*∗*^	43.00 ± 7.84^*∗*^

Note: Sham-operated control (dPBS), *β*-amyloid control (dA*β*), juice mixture + PBS (JPBS), juice mixture + *β*-amyloid (JA*β*), ibuprofen + *β*-amyloid (IBFA*β*). ^*∗*^Significant differences (*p* < 0.05) as compared to after 7 days of A*β* infusion using ANOVA repeated measures. Data are presented as mean ± standard error with *n *=* *8 per treatment group.

**Table 2:** NOR percentage among different rat groups at day 7 and day 14.

NOR percentage (%)/Time of A*β* infusion	dPBS group	dA*β* group	JPBS group	JA*β* group	IBFA*β* group
After 7 days	71.00 ± 7.55	69.00 ± 3.73	64.00 ± 10.77	77.00 ± 3.70	69.00 ± 10.54
After 14 days	44.00 ± 10.23^*∗*^	52.00 ± 9.42^*∗*^	58.00 ± 8.07^*∗*^	69.00 ± 7.54^*∗*^	66.00 ± 10.52^*∗*^

Note: Sham-operated control (dPBS), *β*-amyloid control (dA*β*), juice mixture + PBS (JPBS), juice mixture + *β*-amyloid (JA*β*), ibuprofen + *β*-amyloid (IBFA*β*). ^*∗*^Significant differences (*p* < 0.05) as compared to after 7 days of A*β* infusion using ANOVA repeated measures. Data are presented as mean ± standard error with *n*=8 per treatment group.

## Data Availability

The data sets used and/or analyzed during the current study are available from the corresponding author upon reasonable request.
